# A Study to Assess the Relationship between Attention Deficit Hyperactivity Disorder and Obstructive Sleep Apnea in Adults

**DOI:** 10.7759/cureus.5979

**Published:** 2019-10-24

**Authors:** Sean Hesselbacher, Akshar A Aiyer, Salim R Surani, Alishah A Suleman, Joseph Varon

**Affiliations:** 1 Medicine, Hampton Veterans Affairs Medical Center, Hampton, USA; 2 Pulmonology, Pulmonary Associates, Corpus Christi, USA; 3 Internal Medicine, Texas A&M Health Science Center, Temple, USA; 4 Internal Medicine: Critical Care, Aga Khan University, Tanzania, TZA; 5 Critical Care, United General Hospital, Houston, USA

**Keywords:** obstructive sleep apnea, attention deficit hyperactivity disorder, sleepiness

## Abstract

The association between obstructive sleep apnea (OSA) and attention deficit hyperactivity disorder (ADHD) is well-established in children. However, there is a paucity of literature regarding this association in adults. The aim of this study was to determine if ADHD is more common in adult patients with OSA. All patients referred to a sleep center for sleep evaluation were administered the Adult ADHD Self-Report Scale and diagnostic polysomnogram. The ADHD screen is considered positive if 4 of 6 questions in part A of the screening questionnaire were answered abnormally. The study population consisted of 194 participants, predominantly male (62%), Caucasian (54%), and Hispanic (44%). OSA was identified in 160 (83%) of participants, with 116 (60%) having moderate to severe OSA. The ADHD screen was positive in 37 (19%) of participants. There was no significant association between the severity of OSA and presence of ADHD symptoms. Patients with OSA who screened positive for ADHD had higher Epworth Sleepiness Scale scores than those that did not. These data suggest that ADHD is more prevalent in patients with OSA, but do not demonstrate a relationship between OSA severity and ADHD symptoms. Interestingly, sleepiness is more prominent in patients with ADHD.

## Introduction

Obstructive sleep apnea (OSA) is a common disease with prevalence ranging between 2-7% in the middle age population with apnea hypopnea index (AHI) >5, and is even higher among the elderly and in patients with cardiovascular complications and metabolic syndrome [1-4]. OSA is characterized by recurrent partial or complete airway obstructions during sleep. OSA has been associated with impaired daytime performance, public health consequences, general health complications, and comorbid psychiatric conditions [5-8].

Attention deficit hyperactivity disorder (ADHD) is thought to be one of the most common psychiatric disorders in adults, with an estimated prevalence of 4.4% [9]. Adult ADHD may stem from a new diagnosis, though often persists from childhood. Many of the symptoms of ADHD may mimic those of untreated (or inadequately treated) OSA. Studies have correlated the presence and severity of OSA with ADHD in the pediatric population, though there has been very little work reported in adults. We attempted to examine the associations between ADHD and OSA in a population of adults referred for sleep testing.

The primary aim of this study was to determine if ADHD is more common in adult patients with OSA. Secondary objectives were to determine associations between OSA and ADHD in subgroups of adults, including gender, ethnicity, and age groups, and to determine any associations between ADHD and excessive daytime sleepiness (EDS).

## Materials and methods

Consecutive patients undergoing polysomnography (PSG) at the Baylor College of Medicine Sleep Center in Houston, Texas in 2009 were provided with the ASRS to complete, in addition to standard pre-sleep study paperwork. Inclusion criteria were completion of a diagnostic or split-night sleep study and the ADHD questionnaire, and age >18 years. All records meeting inclusion criteria were selected for review; records were excluded if the patient failed to complete either the ASRS or PSG. All procedures performed in studies involving human participants were in accordance with the ethical standards of the institutional and/or national research committee and with the 1964 Helsinki declaration and its later amendments or comparable ethical standards.

Questionnaires

At the time of PSG testing, each patient was provided questionnaires to complete, including demographic questions (including self-reported age, gender, and ethnicity), the Epworth Sleepiness Scale (ESS), and the Adult ADHD Self-Report Scale (ASRS) [10, 11]. The ASRS is a validated 18-question screen for adult ADHD, comprised of 2 parts (A and B) [12, 13]. Part A consists of 6 questions; the screen is considered positive for ADHD if 4 of these questions are answered abnormally. The sensitivity of this questionnaire for detection of ADHD in adults has been shown to be 68.7%, with a specificity of 99.5%. Presuming a prevalence of 4.4%, this results in a positive predictive value of 86.3% and negative predictive value of 98.6%.

Polysomnography

Sleep studies were performed using attended comprehensive PSG, including recordings of electroencephalogram, electrooculogram, submentalis electromyogram, airflow, respiratory effort, oxygen saturation, anterior tibialis electromyogram, and heart rhythm. Recordings were scored by a technologist manually according to the American Academy of Sleep Medicine Scoring Guidelines and interpreted by a board-certified sleep physician [14].

Statistics

Comparisons between the means of 2 normally distributed groups were performed with the unpaired t-test. Comparisons between 2 non-normally distributed groups were done with the Mann-Whitney U test. The normality of a group distribution was determined using D’Agostino-Pearson omnibus normality test. Two groups of dichotomous variables were compared with the Fisher’s exact test. In defining subgroups for analysis, obesity was defined as a body mass index ≥30 kg/m^2^; ethnicity was broken down into Caucasian and Hispanic groups only, as the others had insufficient numbers to justify subgroup analysis; age was divided at age 50 and 65, which resulted in 3 approximately equal groups (by total numbers); additionally, OSA has been shown to be more severe above age 50 [15]. A P-value of <0.05 was considered statistically significant.

## Results

Demographic and clinical characteristics

A total of 194 unique patients were included in the analyses; 1 patient did not complete the ESS. The mean (± standard deviation) age was 55.6 ±13.9 years; 62% were male. The self-reported ethnicities were: 54% Caucasian, 44% Hispanic, 2% other. OSA of any severity (AHI ≥5) was identified in 160 (82.5%) of the patients, with moderate to severe OSA (AHI ≥15) seen in 116 (59.8%).

Association between OSA and ADHD

A positive result on the ADHD questionnaire (≥4 abnormal responses in part A) was obtained from 37 (19.1%) of the patients. As shown in Table 1, there was no significant association between the severity of OSA and a positive screen on the ADHD questionnaire, in that the proportion of patients screening positive for ADHD were similar among those with absent (AHI <5), mild (AHI 5-14.9), and moderate to severe (AHI ≥15) OSA. Likewise, the presence of a positive ADHD screen was not associated with any significant difference in AHI (25.7±22.8, N=37 vs. 30.6±28.5, N=157; P=0.47). No significant differences were seen when making these comparisons in the subgroups (gender, ethnicity, obesity status, and age group).

**Table 1:** Association between OSA severity and ADHD. *Values are reported as the proportion of patients in each OSA severity group meeting criteria for and abnormal response to the Adult ADHD Self-Report Scale; statistical comparisons were made using Chi square test. *OSA-: obstructive sleep apnea is not present; Mild OSA+: obstructive sleep apnea is present with an apnea-hypopnea index (AHI) 5-14.9 events per hour; OSA(15)+: obstructive sleep apnea is present with an AHI ≥15 events per hour.

	OSA-	Mild OSA+	OSA(15)+	P-value
All (n=194)	0.24	0.14	0.20	0.52
Male (n=120)	0.07	0.12	0.16	0.58
Female (n=74)	0.37	0.16	0.28	0.34
Caucasian (n=105)	0.16	0.08	0.23	0.25
Hispanic (n=85)	0.31	0.21	0.15	0.42
Obese (n=151)	0.30	0.08	0.22	0.32
Non-obese (n=41)	0.14	0.16	0.07	0.71
Age <50 (n=66)	0.31	0.18	0.31	0.70
Age 50-64 (n=69)	0.17	0.24	0.15	0.74
Age ≥65 (n=59)	0.17	0.00	0.13	0.27

Association of ADHD symptoms with sleepiness

**Figure 1:**
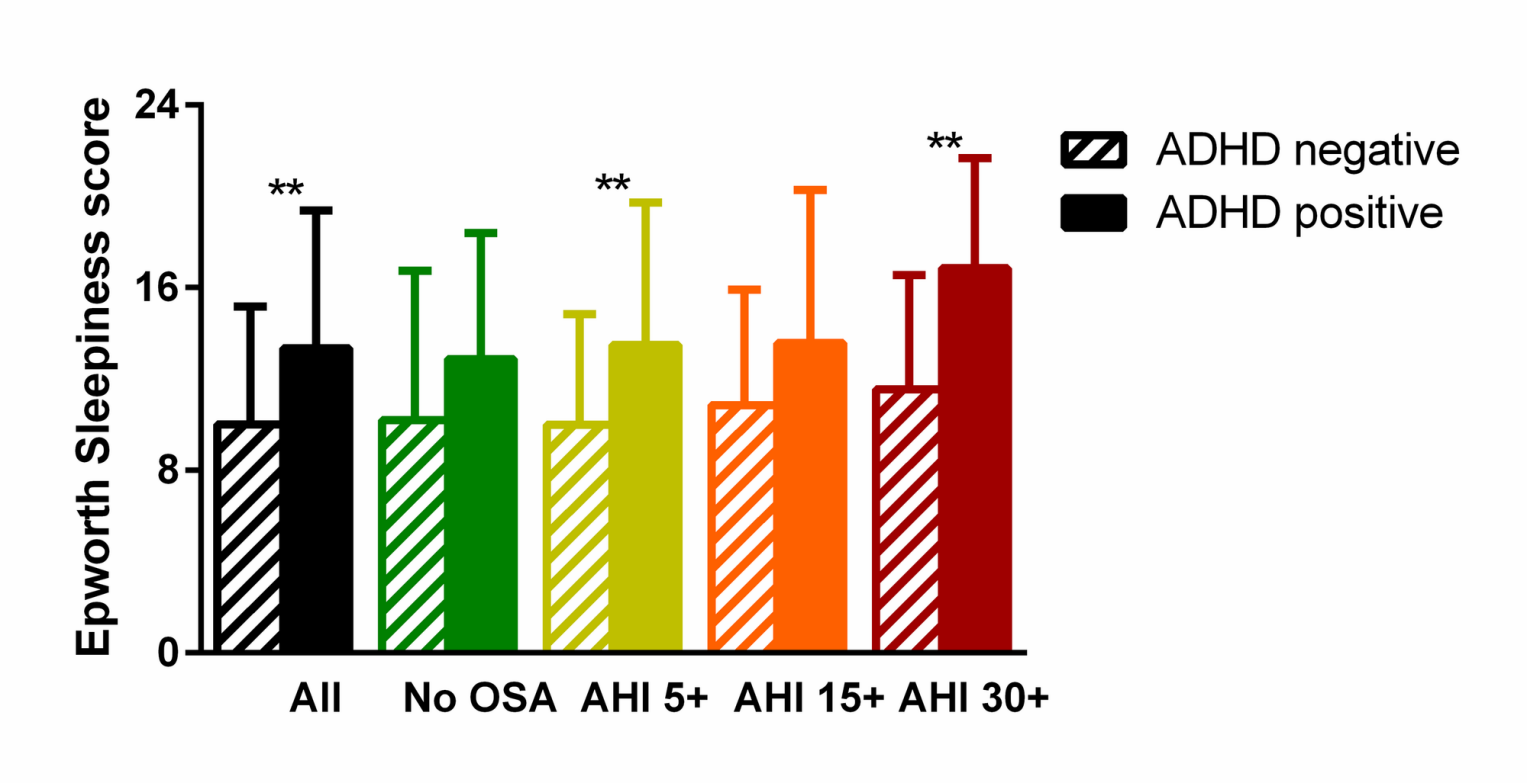
Association between symptoms of attention deficit hyperactivity disorder (ADHD) and sleepiness. In patients with obstructive sleep apnea (OSA) of any severity, a positive screen for ADHD (diagonal lines) was associated with higher scores on the Epworth Sleepiness Scale; this was not seen in patients without OSA (solid colors). **P<0.01 AHI 5+ (yellow): Mild to severe OSA is present with an apnea-hypopnea index (AHI) ≥5 events per hour. AHI 15+ (orange): Moderate to severe OSA is present with an AHI ≥15 events per hour. AHI 30+ (red): Severe OSA is present with AHI ≥30 events per hour.

Scores on the ESS were higher in patients that screened positive for ADHD than those that did not (13.4±6.0, N=37 vs. 10.0±5.1, N=156; P=0.002), as seen in Figure 1 (black bars). A greater proportion of patients screening positive for ADHD also had an abnormal ESS score of >10 (0.70 vs 0.42, P=0.0019). 

## Discussion

Our data shows that in patients with OSA there is a high prevalence of patients screening positive for ADHD (19.1%) - this is substantially higher than the reported prevalence of ADHD in the general population (4.4%). Given the previously reported characteristics of the ASRS, a positive result would be expected in approximately 7.3% of respondents from the general population. Our data did not demonstrate a significant relationship between the severity of OSA and the presence of ADHD symptoms.

The data did show that patients OSA who had ADHD symptoms were, on average, sleepier than those without ADHD symptoms and a greater percentage of ADHD patients had abnormal ESS scores. Previous trials have shown that adults with ADHD tend to have greater subjective sleepiness and our data are consistent with these findings [16]. While none of the questions in part A of the ASRS directly pertain to daytime sleepiness, many can result from sleepiness or poor sleep. It is not hard to imagine someone with EDS having “trouble wrapping up the final details of a project, once the challenging parts have been done” (question 1). Similarly, patients with EDS and sleep disorders often have difficulty remembering appointments or obligations (question 3), and have trouble with organization (question 2) [17, 18]. The other questions relate less directly to EDS. Question 5 (“How often do you fidget or squirm with your hands or feet when you have to sit down for a long time?”) may be answered affirmatively in patients with Restless Legs Syndrome, which has been reported to be more common in patients with OSA [19]. If EDS were to contribute to abnormal answers on even some of these questions, the likelihood of a positive screen would be raised, even in the absence of overt ADHD. Because some questions on the ASRS focus on attention deficits and others on hyperactivity, future research may examine which of the questions, including Part B of the ASRS, are most associated with EDS or other sleep disorders, and whether these truly represent the spectrum of ADHD or simply false-positive results.

Previous reports have commented on the overlap between ADHD symptoms and those attributed to OSA, though these have primarily been in children and adolescents [20, 21]. Our study is consistent with this in that we saw a much higher prevalence of ADHD than the general population of adults. More study would be needed to determine if ADHD assessment is justified in all patients with OSA.

## Conclusions

We saw a high prevalence of ADHD in a population of OSA patients referred for sleep study testing. Despite no relationship to OSA severity, patients with positive ADHD symptoms were generally sleepier than those without ADHD, suggesting a shared common pathophysiologic neurobehavioral influence. The exact mechanisms underlying this shared vulnerability to sleep disruption may need to be further elucidated.
